# Large Cyclability of Elastocaloric Effect in Highly Porous Ni-Fe-Ga Foams

**DOI:** 10.3390/ma17061272

**Published:** 2024-03-09

**Authors:** Muhammad Imran, Mingfang Qian, Xuexi Zhang, Lin Geng

**Affiliations:** 1 School of Materials Science and Engineering, Harbin Institute of Technology, Harbin 150001, China; muhammad.imran@uettaxila.edu.pk (M.I.); genglin@hit.edu.cn (L.G.); 2 Mechanical Engineering Department, University of Engineering & Technology Taxila, Taxila 47050, Pakistan

**Keywords:** ferromagnetic shape memory alloy (FMSMA), elastocaloric effect (eCE), Ni-Fe-Ga, martensitic transition (MT), porous materials, cyclic stability

## Abstract

Solid-state refrigeration based on elastocaloric materials (eCMs) requires reversibility and repeatability. However, the intrinsic intergranular brittleness of ferromagnetic shape memory alloys (FMSMAs) limits fatigue life and, thus, is the crucial bottleneck for its industrial applications. Significant cyclic stability of elastocaloric effects (eCE) via 53% porosity in Ni-Fe-Ga FMSMA has already been proven. Here, Ni-Fe-Ga foams (single-/hierarchical pores) with high porosity of 64% and 73% via tailoring the material’s architecture to optimize the eCE performances are studied. A completely reversible superelastic behavior at room temperature (297 K) is demonstrated in high porosity (64–73%) Ni-Fe-Ga foams with small stress hysteresis, which is greatly conducive to durable fatigue life. Consequentially, hierarchical pore foam with 64% porosity exhibits a maximum reversible ∆*T_ad_* of 2.0 K at much lower stress of 45 MPa with a large *COP_mat_* of 34. Moreover, it shows stable elastocaloric behavior (Δ*T_ad_* = 2.0 K) over >300 superelastic cycles with no significant deterioration. The enhanced eCE cyclability can be attributed to the pore hierarchies, which remarkably reduce the grain boundary constraints and/or limit the propagation of cracks to induce multiple stress-induced martensitic transformations (MTs). Therefore, this work paves the way for designing durable fatigue life FMSMAs as promising eCMs by manipulating the material architectures.

## 1. Introduction

The temperature of superelastic shape memory alloys (SMAs) may vary as isotropic or uniaxial stress is applied/released around the phase transition, which can be described as mechanocaloric effects, including barocaloric and elastocaloric effects (eCE) [1]. Elastocaloric refrigeration based on eCE is a promising cooling technology that can substitute conventional vapor compression, owing to its high compactness, environmental protection and energy-saving potential [2,3]. By utilizing the latent heat generated by the first-order martensitic transformation (MT), many SMAs such as Fe- [4,5], Cu- [6,7], TiNi- [8,9], MnNi- [10,11] and FeNi-based [12,13] systems can exhibit excellent eCE performance, which is characterized by large adiabatic temperature change (∆*T_ad_*) or isothermal entropy change (∆*S_iso_*) [14]. 

In addition, eCE-based refrigeration technology has good miniaturization potential for miniaturized smart devices and systems (e.g., microelectromechanical systems, laser diodes, lab-on-chip systems, etc.) that require active cooling and precise temperature control [15]. For the realization of miniaturized cooling devices, the reduced dimensional forms of elastocaloric materials (eCMs), (for instance, thin films, microwires, ribbons and foams) may also be crucial [16]. However, highly compact refrigeration demonstrators have been developed by taking advantage of TiNi thin films [17], ribbons [18] and wires [19] but their design concepts can be easily modified for miniaturized cooling devices [16,20]. In this context, foams (porous materials) can also be considered as a reduced form of polycrystals because they have small nodes and struts, comparable to the size of the crystal grains. Compared with the bulk counterpart, the foam may also exhibit greater heat transfer capacity because of its large specific surface area [21] and a greater percentage of MT due to the reduction of grain boundary constraints [22]. These characteristics make porous materials very attractive as potential miniaturized elastocaloric refrigerants.

Moreover, ferromagnetic shape memory alloys (FMSMAs [23]), especially Ni-Fe-based systems [24,25], have greater eCE potential for miniaturized refrigeration because they exhibit better eCE performance (∆*T_ad_*/∆*S_iso_*) with small stress hysteresis under lower uniaxial stress during MT than traditional SMAs (TiNi- [26] and Cu-based alloys [27]). However, due to the weaker grain boundary bonding strength (brittleness), their polycrystalline bulk alloys are more prone to fatigue (10–32 superelastic cycles) [24,28], which severely limits their practical eCE applications. Prolonged fatigue life (over 10 million superelastic cycles) with stable eCE performance is essential for the realization of eCE cooling technology. From this perspective, several possible strategies have been successfully adopted to reduce the brittleness of such eCE materials and increase their fatigue life [3]. Among them, the introduction of porosity is also a feasible way to enhance the working stability of bulk materials over repeated MTs via suppressing the grain boundary constraints [29]. Accordingly, our previous research determined that Ni-Fe-Ga single-/hierarchical pore foams with 53% porosity processed by the molten metal infiltration method (using NaAlO_2_ as space holder) can efficaciously improve the cyclic stability (up to 100–194 superelastic cycles) by reducing hysteresis energy loss and decelerating the crack growth rate [24,25].

What is more, SMA foams have been attractive for potential applications in numerous engineering fields, e.g., sensors, actuators, dampers and biomedical devices, where appropriate porosity and pore size are crucial [30]. High-porosity SMA foams have various structural characteristics of pores/struts/nodes at different levels (e.g., macro-, meso- and microlevels), which can impart excellent properties, such as large and stable magnetic field-induced strain (Ni-Mn-Ga foams with 64–76% porosity can be used for millions of thermomechanical cycles) [31,32], damping capacity (NiT foams with 69% porosity) [33], superelasticity (Cu-Al-Mn foams with 66–81%) [34] and biocompatibility (NiTi foams with 30–90% porosity for long-term implantation) [35]. However, by tailoring the material’s architecture, the infiltration method can create a wider range of porosity (64–73% porosity) for Ni-Fe-Ga foams (single-/hierarchical pores). The resulting contraction/elongation incompatibility generated under the influence of uniaxial stresses in various sizes of nodes and struts can be reduced by appropriately applying external stress (compression loading) [36]. Moreover, by limiting the applied stress below the yield strength of the material, low stress hysteresis and high cyclic stability are expected in high-porosity foams [27]. Therefore, further research on high-porosity foams (single-/hierarchical pore foams) with their mesoscopic-scale architectures (pores, struts and nodes) is necessary to optimize their elastocaloric properties.

Here, Ni-Fe-Ga foams (single-/hierarchical pores) with high porosity of 64% and 73% were fabricated via tailoring the material’s architecture route through utilizing the same NaAlO_2_ replication method. The high-porosity (64–73%) Ni-Fe-Ga foams (single-/hierarchical pores) demonstrated the stable superelastic effects at room temperature (297 K). Hierarchical pore foams with 64% porosity showed stable eCE cooling performances (2.0 K under 45 MPa) over 300 loading/unloading cycles (without any degradation) with large *COP_mat_* 34. Thus, the presence of pore hierarchies in porous SMAs obtained via material architectures is a promising route to improve the eCE cyclability of FMSMAs.

## 2. Experimental Details

Ni-Fe-Ga single-/hierarchical pore foams were fabricated using the replication casting approach by utilizing the same NaAlO_2_ (Aladin Bio-Chem Technology Co., Ltd, Shanghai, China) space holder. Detailed descriptions on the preparation of single-/hierarchical pore foams can be found elsewhere [22,25]. In order to obtain high-porosity foams (single-/hierarchical pore foams), chemical corrosion was performed in different acid bath solutions under different sonication times and frequencies (40 kHz) [37]. Our previous work has demonstrated that the dissolution of Ni-Fe-Ga alloy is negligible in acid bath-I (2% HF + 10% H_2_SO_4_), while the dissolution rate is remarkably high in acid bath-II (2% HF + 10% HNO_3_), irrespective of NaAlO_2_ dissolution [24,25]. On account of this, foams with high porosity can be obtained by completely removing NaAlO_2_ and partially dissolved Ni-Fe-Ga alloy. Furthermore, high integral nodes and struts are the main load-bearing components of foams that undergo plastic deformation/hinging during compression. Therefore, by adjusting the ultrasonic treatment time in different acid baths, single-/hierarchical pore foams with various pore sizes and porosities can be obtained without damaging the integral nodes and struts.

Ni-Fe-Ga foams with ~50% porosity can be easily achieved by dissolving the coarse NaAlO_2_ particles in acid bath-I upon prolonged exposure (Spell-I), as depicted in Figure S1 (Supplementary Materials), with the fine particles of NaAlO_2_ still wrapped in thin struts [25]. For this purpose, acid bath-II was used for a short interval of time (10 min) to thin the struts, followed by prolonged exposure (240 min) to acid bath-I (Spell-II). Finally, both acid solutions (Spell-II) were repeated sequentially to obtain Ni-Fe-Ga foams (single/hierarchical pores) with porosity of 64% and 73%. A diamond saw cutter (SYJ-150) was employed to cut the parallelopiped (4 × 4 × 8 mm^3^) foam samples for compression testing. To obtain a single β-phase at room temperature, the foam samples were sealed in a quartz tube under vacuum, then subsequently heated to a temperature 1453 K and kept at this temperature for 5 h, followed by quenching in water.

The chemical compositions (at.%) of the annealed single (Ni_53.5_Fe_19.9_Ga_26.6_) and hierarchical pore (Ni_52.9_Fe_19.2_Ga_27.9_) foams were determined using a scanning electron microscope (SEM, Zeiss Supra 55 SAPPHIRE, Oberkochen, Germany) equipped with an Oxford energy dispersive spectrometer (EDS). The microarchitecture and phases of foams were examined at room temperature by an Olympus PMG3 optical microscope (Olympus, Tokyo, Japan) and PANalytical Empyrean X-ray diffraction (XRD, Malvern Panalytical Ltd, Malvern, United Kingdom) with Cu-Kα radiation (reflection mode). A differential scanning calorimeter (DSC, Discovery-2500 by TA Instruments, Newcastle, DE, USA) was used to characterize the MT temperatures and latent heat with a heating/cooling rate of 10 K/min (interval of 278–350 K), while liquid nitrogen was utilized for low-temperature atmosphere. The superelastic (at low loading speed) and elastocaloric properties (at high loading speed) were measured in compression mode by utilizing the universal testing machines (Instron 5569, Boston, MA, USA for room temperature and Instron 5982, Boston, MA, USA for elevated temperature). 

For each superelastic test, the foam sample was heated to 353 K (>*A_f_*) and held for 5 min, then cooled to the test temperature to record the stress–strain plot with a strain rate of 1.7 × 10^−4^ s^−1^ (under isothermal conditions). For elastocaloric tests, the foam sample was loaded–unloaded under quasi-adiabatic conditions at a higher strain rate of 0.02 s^−1^. The temperature profiles were recorded in situ by infrared (IR) thermal imaging (FLIR A325sc, Wilsonville, OR, USA) with a measurement accuracy ±2% and thermal sensitivity ±0.05 K, where the thermal spectrograms (320 × 240 pixels) were captured at a rate of 30 Hz. To improve the thermal emissivity, the foam samples were covered with a thin layer of graphite spray.

## 3. Results and Discussion

### 3.1. Structural Characterization of High-Porosity Foams

**Figure 1:**
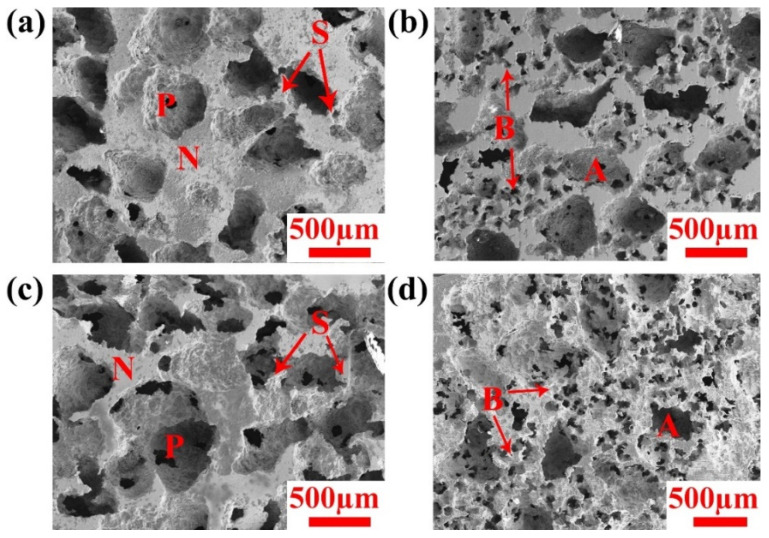
SEM micrographs for Ni-Fe-Ga foams with single-/hierarchical pore architectures and various porosities after chemical etching in acid baths. The nodes (N), struts (S) and pores (P) are marked in single-pore foams with (**a**) 64% porosity and (**c**) 73% porosity. Both large (A) and small (B) pores appear in hierarchical pores foam with (**b**) 64% porosity and (**d**) 73% porosity.

The cross-sectional morphologies of Ni-Fe-Ga foams (single/hierarchical pore) with high porosity of 64% and 73% obtained by SEM are shown in Figure 1a–d. All samples exhibit uniform pore distribution despite differences in pore size and porosity owing to single and hierarchical pore morphology, which means that high integral nodes and struts are completely interconnected with pores. The second set of smaller nodes and struts can be observed in hierarchical pore foams, where small pores (B) are evenly distributed among large pores (A) (see Figure 1b,d). Even foams (single-/hierarchical pore) with high (73%) porosity exhibit a very homogeneous and defect-free architecture. It is also noticed that the size of the pores increases with increasing porosity, which is a result of the dissolution of the alloy. However, all SEM micrographs confirm that the Ni-Fe-Ga foams with high structural homogeneity provide a structural foundation for further exploration of eCE cooling performance under repeated loading.

**Figure 2:**
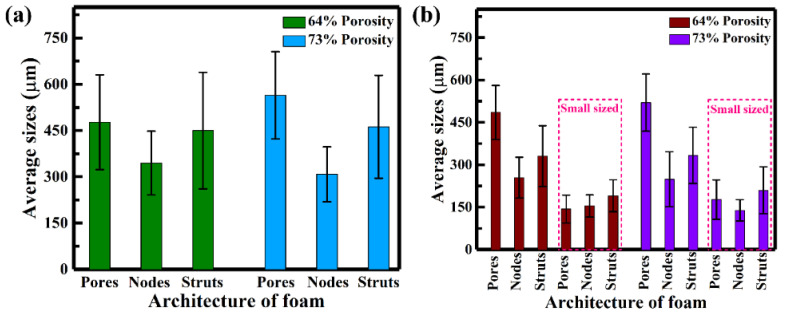
The structural characterization of high-porosity Ni-Fe-Ga foams, showing the average sizes of pores, nodes and struts for (**a**) single-pore foams and (**b**) hierarchical pore foams.

The structural architecture of Ni-Fe-Ga foams (single/hierarchical pore) of various porosities can be characterized by the sizes of pores, nodes and struts, which are measured via the two-dimensional OM images [34], as shown in Figure S2. In addition, Figure 2a demonstrates the architecture of single-pore foam of various porosities (64% and 73%). The single-pore foam with 64% porosity has an average pore size of 477 µm, which is still in good agreement with the designed space holder value (diameter 325–500 µm) [22]. However, the higher porosity (73%) leads to thinner struts, resulting in pores of larger size (564 µm). The average node size is found to be largely dependent on the pore size. When the porosity is increased from 64% to 73%, the node size effectively decreases from 344 µm to 308 µm with increasing pore size. The average strut size of single-pore foam is in the range of 450–462 µm, while its width decreases remarkably with increasing porosity from 56 to 52 µm.

Figure 2b shows the structural architecture of hierarchical pore foam (for higher porosity of 64% and 73%). The hierarchical pore foams are produced from dual-sized space holder particles (diameters of 325–500 µm and 75–90 µm) [25], which correspond to pore sizes of specific porosities. It can be seen that the size of large pores has little effect in increasing the porosity (64–73%), as it varies from 485 to 520 µm, although the size of smaller pores changes dramatically (143–176 µm) because of the smaller interstitial space between the smaller pores and thinner struts. As the porosity increases from 64 to 73% porosity, larger and smaller nodes appear in the range of 254–249 µm and 154–138 µm, respectively. The average size of the larger (330–333 µm) and smaller struts (190–209 µm) shows a negligible effect with increasing porosity, while their widths change significantly (107–59 and 56–20 µm for larger and smaller strut widths, respectively). 

### 3.2. Martensitic Transformation Behavior

The DSC curves of Ni-Fe-Ga foams (single-/hierarchical pores) during cooling and heating are shown in Figure S3. The exothermic and endothermic peaks associated with forward and reverse MTs for both foams lie between 275 and 295 K. It can be seen that the forward (*M_s_* and *M_f_*) and reverse (*A_s_* and *A_f_*) MT temperatures for single- (284, 276, 285 and 292 K) and hierarchical pore (282, 276, 283 and 291 K) foams are determined via the tangent extrapolation method. Both single- and hierarchical pore foams exhibit austenite β-phase around room temperature, which is also confirmed by the XRD patterns, as depicted in Figure S4. Such MT behavior around room temperature makes them practicable for room temperature eCE refrigeration.

Furthermore, the thermally induced entropy change (∆*S_tr_*) associated with MT can be computed by ∆*S_tr_* = *Q_rev_/T*_0_ through DSC analysis, where *Q_rev_* is the latent heat and *T*_0_ is the equilibrium temperature, expressed as (*A_s_* + *A_f_*)/2) [38]. The maximum value of ∆*S_tr_* on the heating sequence is computed to be ~10.5 J/kg·K for both types of foams. The obtained ∆*S_tr_* = 10.5 J/kg·K value is comparable to other elastocaloric SMAs, such as Ni_55.1_Fe_16.2_Ga_28.7_ ribbon (12 J/kg·K) [39] and Ni_47.4_Ti_52.6_ wire (12 J/kg·K) [40]. The maximum theoretical adiabatic temperature change (∆*T_th_*) during reverse MT can be determined indirectly through the relation ∆*T_th_* = *T*·∆*S_tr_*/*C_p_* [41], where *C_p_* is the specific heat capacity (*C_p_* = 325 J/kg·K [24]). The upper bound on ∆*T_th_* for both foams (single-/hierarchical pore) is estimated to be ~9.5 K at 297 K, which is comparable to other well-studied eCE cooling materials, for instance, Ni-Fe-Ga polycrystal (7.2 K) [24], Fe-Pd single crystal (3.1 K) [4] and natural rubber (4 K) [42]. Such large ∆*S_tr_* and ∆*T_th_* in current Ni-Fe-Ga foams indicate a considerable eCE cooling potential. 

### 3.3. Superelastic Response

**Figure 3:**
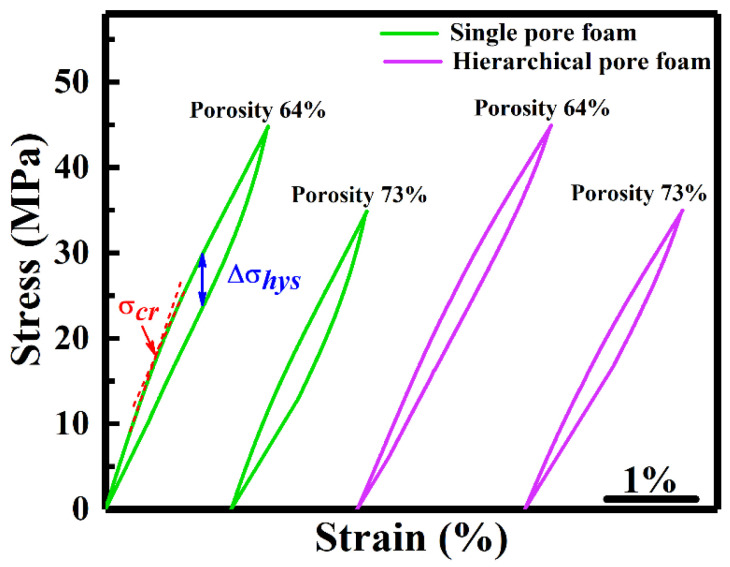
Compressive superelastic stress–strain response in highly porous Ni-Fe-Ga foams at 297 K (above *A_f_*) under the strain rate 1.7 × 10^−4^ s^−1^.

Figure 3 shows the compressive superelastic response of Ni-Fe-Ga foams at 297 K (above *A_f_*), with a loading/unloading strain rate of 1.7 × 10^−4^ s^−1^. In order to avoid crack initiation in high-porosity foams, the maximum nominal stress is limited to 45 and 35 MPa for foams with 64% and 73% porosity, respectively. Considering the presence of porosity in Ni-Fe-Ga foams, the actual stress required to induce MT is higher, i.e., (45 MPa/(1–64%)) = 125 MPa and (35 MPa/(1–73%)) = 130 MPa. As can be seen, both foams (single-/hierarchical pore) display fully reversible superelasticity, with maximum recoverable strain of 1.9% (64% porosity) and 1.6% (73% porosity) for single-pore foam and 2.3% (64% porosity) and 1.8% (73% porosity) for hierarchical pore foam, resulting from stress-induced MT. 

It should be noted that the critical stress (*σ_cr_*) for the onset of MT is quite low in our high-porosity foams, i.e., 18 MPa (64% porosity) and 11 MPa (73% porosity) for single-pore foams and 13 MPa (64% porosity) and 9.6 MPa (73% porosity) for hierarchical pore foams, which are lower than the values reported in previous studies such as Ni_34_Ti_54_Cu_12_ film (200 MPa) [43], Ni_53.5_Fe_19.9_Ga_26.6_ foam (32 MPa) [24], Ni_50_Fe_19_Ga_27_Co_4_ single crystal (90 MPa) [44], Ni_55_Mn_18_Ga_26_Ti polycrystal (80 MPa) [45] and Ni_43_Mn_47_Sn_10_ textured polycrystal (106 MPa) [46]. This reduced critical stress should be attributed to the minimization of grain boundary constraints in high-porosity foams, which favors repetitive stress-induced MT.

Furthermore, stress hysteresis (Δ*σ_hys_*) is the stress difference between the loading and unloading plots of the compressive stress–strain response, shown in Figure 3, arising from the inherent dissipative heat of internal friction during stress-induced MT. The interaction between existing and nucleated multivariant MT and matrix strength leads to the generation of frictional dissipation, which ultimately promotes Δ*σ_hys_* in SMAs during stress-induced MT [47]. As a consequence, a larger Δ*σ_hys_* generates entropy, larger mechanical work and temporary residual strain, which reduce the reversibility, cooling efficiency and fatigue life of eCE materials [48]. From this perspective, small Δ*σ_hys_* is more desirable in eCE cooling materials for durable eCE cooling technology. Smaller Δ*σ_hys_* of ~6 MPa and ~4.5 MPa are found in our high porosity Ni-Fe-Ga single-pore (64% and 73% porosity) and hierarchical pore (64% and 73% porosity) foams, respectively (Figure 3). This small Δ*σ_hys_* is mainly attributed to the reduction in frictional work (dissipation of energy) during stress-induced MT due to the suppression of the grain boundary constraints through pores. 

**Figure 4:**
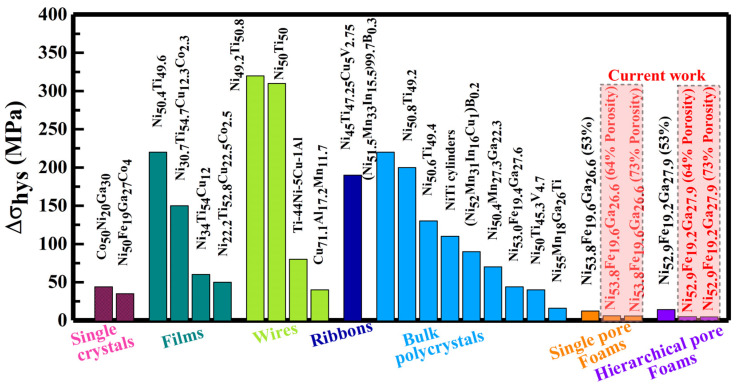
Stress hysteresis (Δ*σ_hys_*) in high-porosity Ni-Fe-Ga foams (single-/hierarchical pore) [24,25] and some well-studied eCE SMAs such as single crystals [44,49], films [43,50,51,52], wires [27,53,54,55], ribbon [56] and textured- [45,57]/non-textured polycrystals [24,58,59,60,61,62,63].

Figure 4 summarizes Δ*σ_hys_* of the present high-porosity Ni-Fe-Ga foams and some well-studied eCE SMAs. It can be seen that the Δ*σ_hys_* of the present high-porosity foams (single-/hierarchical pore) is significantly smaller than those of single crystals [44,49], films [43,50,51,52], wires [27,53,54,55], ribbon [56] and textured [45,57]/non-textured polycrystals [24,58,59,60,61,62,63]. Additionally, Ni-Fe-Ga FMSMA with high-porosity foams (9.6–18 MPa) exhibits excellent small Δ*σ_hys_* compared to Ni_50_Fe_19_Ga_27_Co_4_ single crystals (35 MPa) [44], Ni_55_Fe_16_Ga_29_ polycrystals with single β-phase (44 MPa) [24] and Ni_53.2_Fe_19.4_Ga_27.4_ polycrystals with dual-phase (β + γ) (60 MPa) [64].

It is worth mentioning that the micropillars/wires (same as microsized struts/nodes in foams) show reduced Δ*σ_hys_* in overcoming the interfacial motion resistance between MT (austenite ↔ martensite) [65]. Thus, reduced Δ*σ_hys_* is achieved in high-porosity foams due to reduced grain boundary constraints by introducing pores. Furthermore, compared to the thick nodes/struts in single-pore foams, the thin nodes/struts in hierarchical pore foams display a small dissipation of elastic strain energy associated with plastic deformation [66]. Therefore, the hierarchical pore foams (4.5–4.7 MPa) show a better reduction in Δ*σ_hys_* compared to single-pore foams (5.8–6 MPa).

### 3.4. Evaluation of Elastocaloric Effect (eCE) and Its Cyclic Stability

To further investigate eCE in high-porosity Ni-Fe-Ga foams, the temperature variation (Δ*T_ad_*) during stress-induced MT was monitored using an IR camera at a high strain rate of 0.02 s^−1^ (during loading–unloading) to ensure a quasi-adiabatic state [51]. Foam (both single- and hierarchical pore) samples with 64% and 73% porosity were loaded at target stresses of 45 MPa and 35 MPa, respectively, then held for 60 s to ensure that sample temperature equilibrated with ambient temperature; finally, the target stress was rapidly removed. The time-dependent Δ*T_ad_* profiles of Ni-Fe-Ga foams (both single- and hierarchical pore) measured at room temperature (297 K) are shown in Figure S5. As can be seen, Ni-Fe-Ga foams with high porosity (64% and 73%) exhibit good reversibility of Δ*T_ad_* during the loading/unloading process. The maximum Δ*T_ad_* achieved in hierarchical pore foams is 2.0 K/−2.0 K and 1.0 K/−1.0 K (during loading/unloading) for 64% and 73% porosity, respectively. Similarly, 1.4 K/−1.4 K and 0.6/−0.7 K (during loading/unloading) are obtained in single-pore foams with 64% and 73% porosity. 

It is worth mentioning that the experimental values of Δ*T_ad_* are far less than the theoretical values (Δ*T_th_*) measured via DSC analysis. The imperfect adiabatic conditions and incomplete MT (under lower stress) [58] are responsible for the small Δ*T_ad_* during stress-induced MT. Moreover, non-uniform temperature distribution occurs in foam due to its unique architecture, namely, nodes, struts and pores of various sizes, which refers to the fraction of material involved in elastocaloric activity, rather than the entire material, such as in the bulk material. To analyze the combined effects of struts, nodes and pores, we measured an “average temperature reading” from the entire structure by averaging the node and strut temperatures and discarding the pore regions [24]. Furthermore, the struts and nodes are subjected to bending/hinging and axially/shear deformation during the compression of open-pore foam [67], as shown in Figure S6. Axially loaded foam parts undergo shear deformation, resulting in greater stress-induced MT and eCE activity in the foam compared to the bending or plastically hinged parts. However, the bending deformation may lead to stress-induced MT and eCE activity in various materials [68,69], but the bended parts of foam do not contribute to eCE activity. As a consequence, uneven stress-induced MT may occur in various parts of foam, which results in lower eCE activity (i.e., smaller Δ*T_ad_* compared to Δ*T_th_* assessed by DSC data). Hence, if we consider the presence of porosity in our single-/hierarchical pore foams, then the actual temperature variation (Δ*T_actual_* = Δ*T_ad_*/porosity) may be higher than the calculated values, i.e., 5.6 K and 2.8 K for hierarchical pore foams with 64% and 73% porosity, respectively. Likewise, the values were 3.9 K and 2.2 K for single-pore foams with 64% and 73% porosity, respectively. However, the Δ*T_ad_* obtained in Ni-Fe-Ga foams (single-/hierarchical pore with 64% and 73% porosity) can still be compared with other well-explored eCE SMAs, for instance, Fe_68.8_Pd_31.2_ (~2.1 K) [4] and Ni_59_Fe_18_Ga_27_Co_6_ [70] single crystals; (Ni_51.5_Mn_33_In_15.5_)_99.7_B_0.3_ (~2.0 K) [58] and Ni_55_Mn_7_Ga_27_Fe_11_ (2.4 K) [11] polycrystals and Ni_51.5_Ti_48.5_ (4.1 K) [71] and Ni_46_Ti_50_Fe_2_ (2.7 K) [72] traditional alloys.

**Figure 5:**
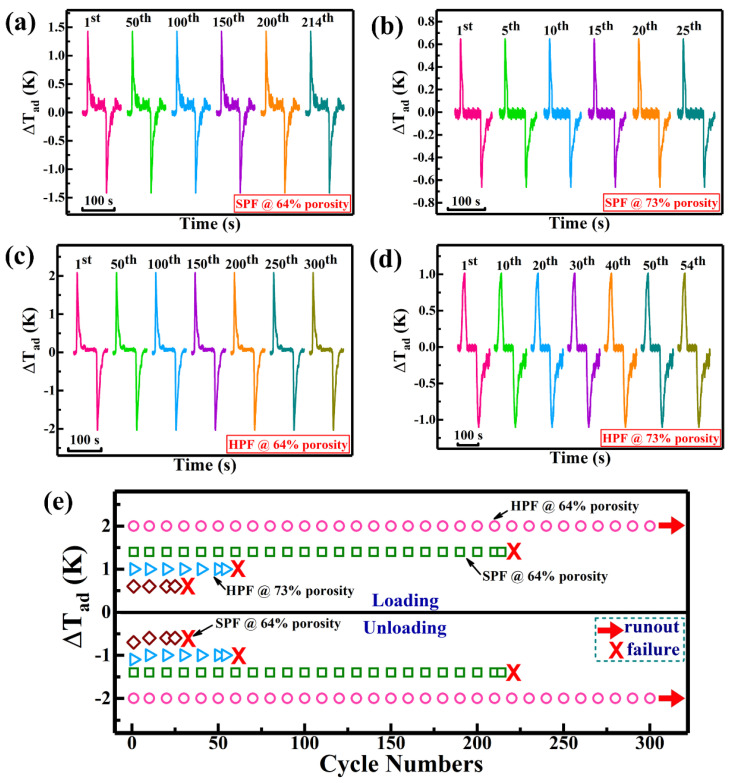
Time-dependent ∆T_ad_ in high-porosity Ni-Fe-Ga foams measured at 297 K with a strain rate of 0.02 s^−1^. (**a**) Single-pore foams (SPF) with 64% porosity. (**b**) Single-pore foams (SPF) with 73% porosity. (**c**) Hierarchical pore foams (HPF) with 64% porosity. (**d**) Hierarchical pore foams with 73% porosity. (**e**) Comparison of ∆T_ad_ during multiple cycles for single-/and hierarchical pore architecture foams with different porosities (64% and 73%).

As is well known, the cyclic stability of eCE is an essential factor for evaluating the solid-state cooling systems, where eCE requires high fatigue life for its commercialization. Therefore, eCE cyclic stability of Ni-Fe-Ga foams with high porosity was investigated at 297 K (slightly higher than *A_f_*) and a high strain rate (during loading–unloading) of 0.02 s^−1^. Figure 5 shows the time-dependent Δ*T_ad_* patterns of Ni-Fe-Ga foams (single-/hierarchical pore) with different porosities (64% porosity under 45 MPa and 73% porosity under 35 MPa). Ni-Fe-Ga single-pore foam with 64% porosity exhibits good cyclic stability over 214 cycles with a reversible Δ*T_ad_* of 1.4 K/−1.4 K during loading/unloading, as shown in Figure 5a, while the Ni-Fe-Ga single-pore foam with 73% porosity can only sustain 25 cycles with a reversible Δ*T_ad_* of 0.6 K/−0.6 K during loading/unloading processes, as depicted in Figure 5b. Compared with the single-pore foams, the hierarchical pore foams with 64% and 73% porosity show enhanced eCE cyclic stability, as demonstrated in Figure 5c,d. Ni-Fe-Ga hierarchical pore foam with 64% porosity exhibits excellent eCE cyclic stability over 300 cycles with a reversible Δ*T_ad_* of 2.0 K/−2.0 K during loading/unloading. Even after 300 cycles, no obvious deterioration of the specimen (hierarchical pore foam with 64% porosity) is detected. However, the hierarchical pore foam with 73% porosity maintains its cyclic stability for up to 54 cycles with a reversible Δ*T_ad_* of 1.0 K/−1.0 K during loading/unloading processes. The extraordinary eCE cyclic stability and superb fatigue resistance in hierarchical pore foams (with 64% porosity) may be attributed to the narrow stress hysteresis at small nominal stress, low critical stress to induce MT and high resistance to crack initiation/propagation ability. Moreover, the corresponding stress–strain plots of Ni-Fe-Ga foams with different porosities are shown in Figure S7, in which the hierarchically porous foam with 64% porosity displays excellent eCE cyclic stability for 300 cycles with no significant drop in critical transition stress.

Polycrystalline Ni-Fe-Ga bulk alloys (single β-phase) are inherently brittle and exhibit low grain boundary bond strength, which are prone to intergranular fracture [64]. The presence of grain boundaries (crystallographic defect) inhibits MT under repeated stress cycles, leading to stress concentration/crack generation at grain boundaries, resulting in lower ∆*T_ad_* than theoretical and poor reversibility/cyclic stability of elastocaloric response. Hence, Ni-Fe-Ga bulk alloys (single β-phase) display limited eCE cyclic stability, i.e., Ni_54_Fe_19_G_27_ (5 K over 10 cycles) [28] and Ni_53_Fe_19.4_G_27.6_ (4 K over 32 cycles) [24]. Nevertheless, the grain boundary constraints of polycrystalline bulk alloys can be effectively suppressed by porosity (i.e., introducing pores) through isolating the neighboring grains. Due to high damping capacity, the pores are conducive to minimizing the stress concentrations at grain boundaries during repeated stress cycles, where pores can serve as strain-incompatible buffers (against the effects of different stresses on nodes and struts of different sizes), even though the grains span more than one strut/node [73]. 

Moreover, large Δ*σ_hys_* can be found in bulk alloys (35 MPa) during MT (see Figure 4) due to the extremely limited volume expansion in adjacent grains. In contrast, foams (single-/hierarchical pores) exhibit lower Δ*σ_hys_* (4.5–6 MPa) during MT through isolating the adjacent grains and, thus, reducing grain boundary constraints. Therefore, single-pore foams with 64% porosity maintain better eCE cyclic stability over 214 cycles, where greater stress-induced MT may occur in small structures (struts and nodes less than the size of grains) that are less restricted by adjacent grains. Thus far, single-pore foams with 64% porosity display enhanced eCE mechanical stability under repeated loading/unloading through less-constrained MT and reduced grain boundary constraints, which may be attributed to the lower critical stress and narrower stress hysteresis. But the multiple cycles during loading/unloading lead to structural fatigue of single-pore foams due to crack formation, which eventually leads to specimen fracture after several cycles (214 cycles). By contrast, single-pore foams with 73% porosity have low integrated nodes and struts that lose material integrity through rapid crack growth. As a consequence, a limited eCE cyclic stability (25 cycles) is achieved in high-porosity (73% porosity) single-pore foam during repeated MTs. 

In general, fatigue involves the initiation and propagation of cracks that ultimately lead to fracture. To prolong fatigue life, crack propagation lasts longer than crack initiation. This means that lower crack growth rates are critical to maintaining material integrity over multiple loading/unloading MT cycles. It must be emphasized that pores are the crack-arresting sites that prevent crack propagation, provide greater mechanical stability and inhibit damage to foam specimens. Therefore, the eCE cyclic stability/durable fatigue life in hierarchical pore foams can be improved by decelerating the crack propagation since the initiation/propagation of cracks is much slower in thin struts/nodes of hierarchical pore foam compared to thick struts/nodes of single-pore foam [37]. Consequentially, the hierarchical pore foams having 73% porosity can withstand only 54 cycles owing to low integral nodes/struts, while the excellent eCE cyclic stability over 300 cycles is obtained in hierarchical pore foams with 64% porosity (see Figure 5e). Such enhanced eCE cyclic stability can be ascribed to the hierarchical pore structure (having 64% porosity), where the dimensions of the struts/nodes are equal to or smaller than the grain size, which facilitates the unimpeded movement of the martensitic variants during repeated loading/unloading cycles. 

However, the present hierarchical pore foam with 64% porosity demonstrates the highest cyclic stability (300 cycles under 45 MPa) for Ni-Fe-Ga (single β-phase) polycrystalline alloys, i.e., Ni_54_Fe_19_Ga_27_ bulk alloy (10 cycles under 133 MPa) [28], Ni_53_Fe_19.4_G_27.6_ bulk alloy (32 cycles under 130 MPa) [24], Ni_53.5_Fe_19.9_Ga_26.6_ single-pore foam with 53% porosity (100 cycles under 60 MPa) [24] and Ni_52.9_Fe_19.2_Ga_27.9_ dual-pore foam with 53% porosity (194 cycles under 60 MPa) [25]. Moreover, the current cyclic stability (300 cycles) even seems superior to other well-studied eCE materials. For instance, Ni_54_Fe_19_Ga_27_ (β + γ) (100 cycles under 170 MPa) [28], Ni_51.5_Mn_33_In_15.5_ (23 cycles under 300 MPa) [58], Ni_55_Mn_18_Ga_27_ (19 cycles under 350 MPa) [74], Ni_46_Fe_32_Mn_18_Al_4_ (30 cycles under 300 MPa) [75], (Ni_52_Mn_31_In_13_Cu_1_)B_0.2_ (100 cycles under 220 MPa) [59], Ni_50.4_Mn_27.3_Ga_22.3_ (100 cycles under 200 MPa) [57], Ni_50_Mn_30_Ga_20_ (200 cycles under 400 MPa) [76], Co_50_Ni_20_Ga_30_ single crystals (100 cycles under 150 MPa) [49] and some traditional SMAs i.e., Cu_68_Zn_16_Al_16_ (100 cycles under 275 MPa) [77], Cu_70.4_Al_17.2_Mn_12.4_ (51 cycles under 800 MPa) [78] and Ni_50.8_Ti_49.2_ (100 cycles under 600 MPa) [62]. 

Furthermore, stress-induced MT is more pronounced and uniform in thin struts/nodes in hierarchical pore foam compared to thick struts/nodes in single-pore foam, implying a higher proportion of material affected by stress-induced MT in hierarchical pore foam. Therefore, the plastic deformation is limited to a small range, i.e., micro-sized struts/nodes are subjected to MT, thereby improving the ductility of the small-sized material. In addition, the hierarchical pore foams show significantly reduced hysteresis and increased reversibility, as thicker nodes can absorb more elastic energy than thin struts/nodes during repeated stress-induced MT under bending/hinging of parts. In addition, the improved cyclic stability (in high-porosity hierarchical pore foam) may also be ascribed to the minor loop process [79] at low stress (35–45 MPa), which is only responsible for inducing the MT portion. Such a minor loop process does not require any extra energy to nucleate/develop the new martensitic variants during repeated MT, which is conducive to enhancing the reversibility and cyclic stability. Thus far, it is interesting to reveal whether the cyclic stability of solid-state refrigeration can also be improved via introducing porosity (where the size of struts/nodes are less than or equal to size of grains) in other FMSMAs, which is still an open question in this domain.

To evaluate high-efficiency eCMs for practical application in solid-state cooling technology, *COP_mat_* = *Q*/*W* is introduced, where *Q* is the cooling capacity computed according to $∆TC_{p}\rho$ and *W* is the input work by integrating the enclosed area of the stress–strain plot (see Figure S7). Consequentially, the maximum *COP_mat_* for Ni-Fe-Ga single-pore foams of different porosities are calculated to be 21 (64% porosity) and 12 (73% porosity) at 45 MPa and 35 MPa, respectively. Accordingly, the maximum *COP_mat_* for Ni-Fe-Ga hierarchical pore foams of different porosities are calculated to be 34 (64% porosity) and 23 (73% porosity) at 45 MPa and 35 MPa, respectively. Due to the relatively low stress hysteresis, the present hierarchical pore foam with 64% porosity can demonstrate a very high *COP_mat_* value of 34. Such a value is higher than those in some well-known eCMs, for instance, Ni_55_Mn_18_Ga_26_Ti_1_ polycrystalline bulk (25.6) [45], Ni_55_Mn_18_Ga_26_ polycrystalline bulk (21.9) [74], Ni_49.2_Ti_40.8_Cu_10_ polycrystalline bulk (13) [80], Ni_50_Fe_19_Ga_27_Co_4_ single crystalline (14) [44] and Ni_53.2_Fe_19.4_Ga_27.4_ polycrystalline bulk (14.5) [64]. Thus, high-porosity Ni-Fe-Ga foams are expected to provide durable fatigue life and high *COP_mat_* at much lower stress compared to polycrystalline bulk alloys, which makes them promising eCE materials for miniaturized solid-state refrigeration. A complete schematic of the results obtained for high-porosity Ni-Fe-Ga foam can be observed in Figure S8.

For the practical utilization of eCE-based solid-state cooling technology, the eCMs have to withstand millions of cycles in practical devices (refrigerators or heat pumps). For the sake of long-term service, the eCMs should be fatigue-resistant (to both structural and functional fatigue) without the loss of cooling/heating power. Although improved eCE cyclic stability has been demonstrated in the current hierarchical pore foam (64% porosity), it remains unsatisfactory (10^7^ cycles) for commercial and industrial applications. Therefore, our work here simply opens a new avenue to optimize the reversibility/reproducibility of superelasticity and the associated eCE performance by utilizing high-porosity SMAs as promising eCMs. However, there is still a lot of room to improve the eCE cyclic stability of Ni-Fe-Ga foams, for instance, by introducing interstitial secondary ductile phase, utilizing (Cu, Co or Gd) quaternary alloys and manipulating different pore architectures.

## 4. Conclusions

We explored eCE cooling performances of mesoscale high porosity (64–73%) Ni-Fe-Ga foams (single-/hierarchical pores) prepared using a replication casting technique through tuning the material’s architecture, aiming to open a new avenue to exploit porous eCMs in elastocaloric-based solid-state refrigeration. The main outcomes were drawn as follows:

1. High-porosity (64–73%) foams at the mesoscopic level were successfully prepared by chemical etching in two acid baths (2% HF + 10% H_2_SO_4_ and 2% HF + 10% HNO_3_) under ultrasonic treatment. One needs to be very careful during the etching process, otherwise the mesoscopic level of the struts/nodes may be damaged, which are the main load-bearing structures in the Ni-Fe-Ga foams;
2. Both single- and hierarchical pore foams with high porosities (64–73%) exhibit reversible superelasticity (1.6–2.3% recoverable strain) at room temperature (297 K) with quite small stress hysteresis (9.6–18 MPa), which is favorable for reproducing multiple superelastic cycles under room temperature elastocaloric cooling;
3. Hierarchical pore foam with 64% porosity yields a maximum reversible ∆*T_ad_* of 2.0 K at a compressive stress of 45 MPa with a strikingly large *COP_mat_* of 34, which is much higher than those in some well-known eCMs;
4. Hierarchical pore foam with 64% porosity demonstrates stable elastocaloric behavior with insignificant degradation over >300 cycles (where ∆*T_ad_*~2.0 K remains constant), which is the largest number reported in Ni-Fe-Ga (β-phase) polycrystalline FMSMA;
5. The outstanding reversible/reproducible superelastic and elastocaloric behavior in the presented hierarchical pore foams (with 64% porosity) can be explained well by the existence of pore hierarchies, which are processed by tailoring the approach to the material’s architecture. Such pore hierarchy effectively suppresses grain boundary constraints and/or limits the propagation of cracks, thereby reducing stress hysteresis and lowering the critical stress to induce multiple stress-induced MTs.

## Data Availability

The raw data supporting the conclusions of this article will be made available by the authors on request.

## Supplementary Materials

The following supporting information can be downloaded at: https://www.mdpi.com/article/10.3390/ma17061272/s1, Figure S1: A plot between the foam porosity and sonication time for Ni-Fe-Ga (single-/hierarchical pore) foams which are immersed in mixed acids under sonication. Figure S2: The polished cross-section of optical micrographs for Ni-Fe-Ga foams with single-/hierarchical pore architectures and various porosities after chemical etching in mixture of acids. The nodes (N), struts (S) and pores (P) are marked in single pore foam with (a) 64% porosity and (b) 73% porosity. Accordingly, the smaller nodes (n), struts (s) and pores (p) are marked in hierarchical pore foam with (a) 64% porosity and (b) 73% porosity. Figure S3: The DSC plots of cooling and heating for single and hierarchical pore foams represent the MT temperatures. Figure S4: XRD patterns for single and hierarchical pore foams at room temperature exhibiting cubic austenite structure. Figure S5: Time-dependence ∆T_ad_ in single-/hierarchical pore architectures Ni-Fe-Ga foams with high porosities 64% and 73% at room temperature. Figure S6: A honeycomb with hexagonal cells represents the open pore foam architecture, which showing the deformation mechanism in simple geometry of foam. Figure S7: Cyclic stress-strain profiles in single-/hierarchical pore architectures Ni-Fe-Ga foams with high porosities 64% and 73% at room temperature. Figure S8: A complete schematic diagram about the large cyclic stability of High porosity Ni-Fe-Ga foams.
