# Supplementary material for: Large Cyclability of Elastocaloric Effect in Highly Porous Ni-Fe-Ga Foams
Source: Materials (Basel). 2024 Mar 9;17(6):1272. doi: 10.3390/ma17061272 (PMC10972506; doi:10.3390/ma17061272)
Supplement: Supplementary file 1 [file materials-17-01272-s001.zip › materials-2904741-supplementary.pdf]

Supplementary Materials

Large cyclability of elastocaloric effect in highly porous Ni-Fe-Ga foams

Muhammad Imran[1,2], Mingfang Qian[1]*, Xuexi Zhang[1]* and Lin Geng

[1]School of Materials Science and Engineering, Harbin Institute of Technology, Harbin 150001, China

[2]Mechanical Engineering Department, University of Engineering & Technology Taxila, Taxila 47050, Pakistan

*Corresponding authors. Tel/Fax: 86-451-86413921, E-mail: mingfang.qian@hit.edu.cn, xxzhang@hit.edu.cn

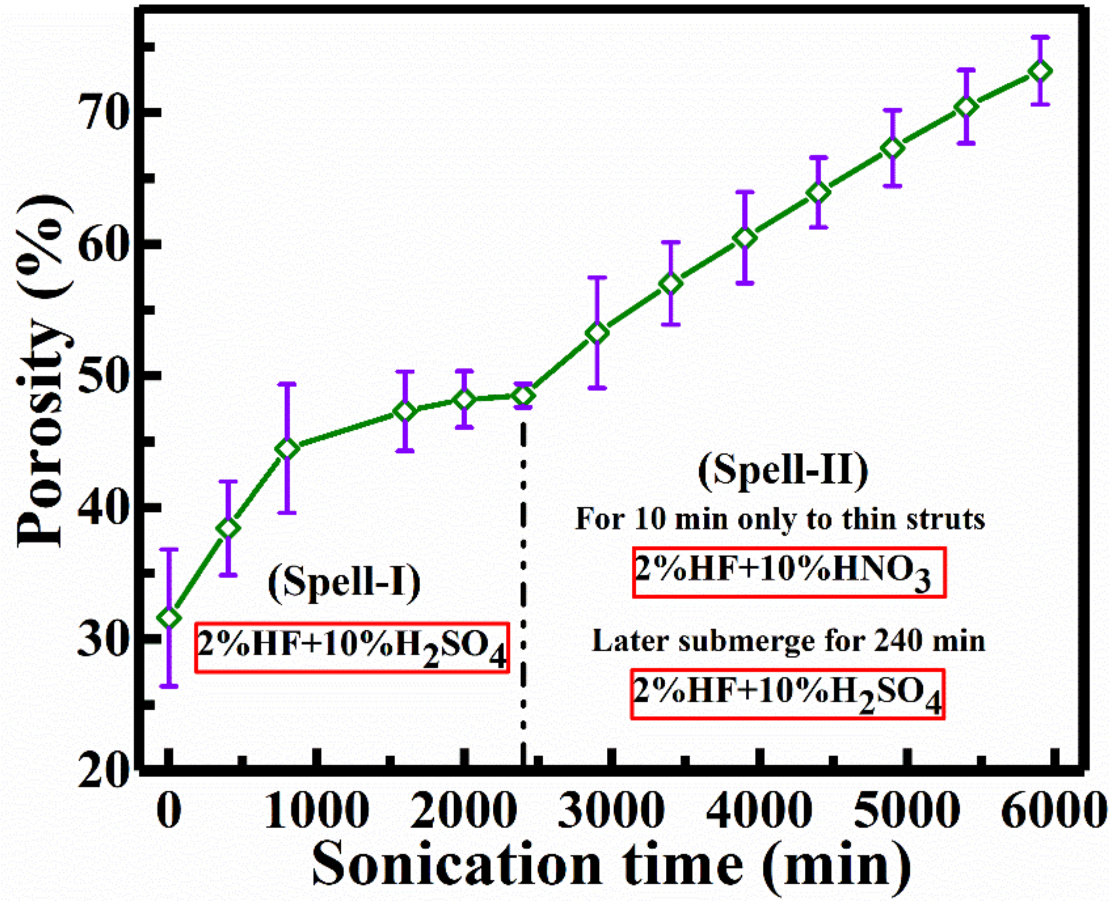

Figure S1. A plot between the foam porosity and sonication time for Ni-Fe-Ga (single-/hierarchical pore) foams which are immersed in mixed acids under sonication.

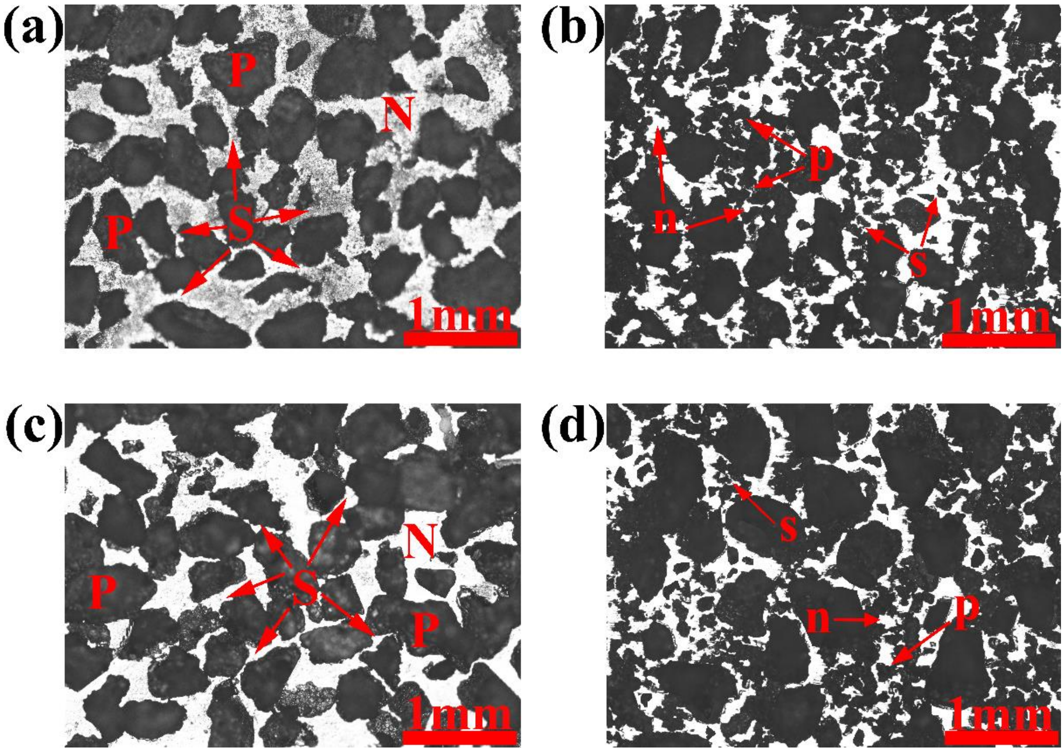

Figure S2. The polished cross-section of optical micrographs for Ni-Fe-Ga foams with single-/hierarchical pore architectures and various porosities after chemical etching in mixture of acids. The nodes (N), struts (S) and pores (P) are marked in single pore foam with (a) 64% porosity and (b) 73% porosity. Accordingly, the smaller nodes (n), struts (s) and pores (p) are marked in hierarchical pore foam with (a) 64% porosity and (b) 73% porosity.

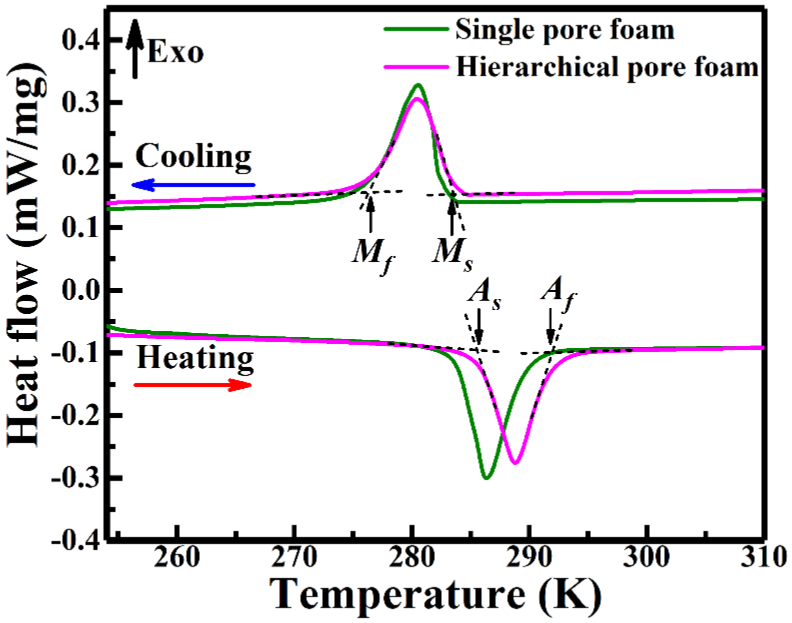

Figure S3. The DSC plots of cooling and heating for single and hierarchical pore foams represent the MT temperatures.

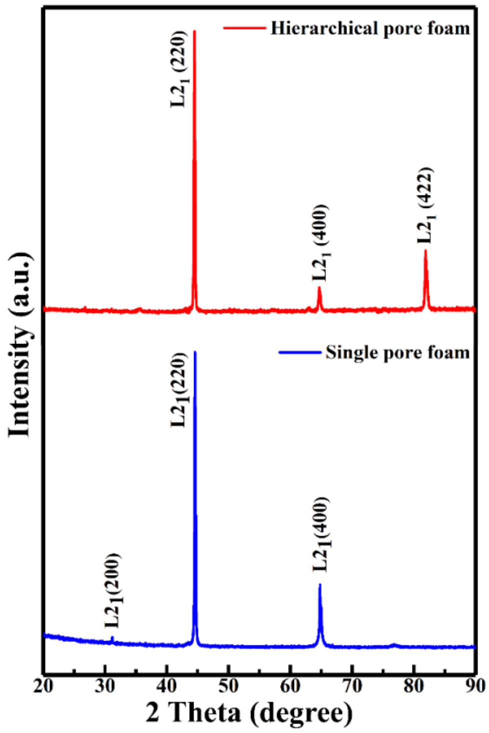

Figure S4. XRD patterns for single and hierarchical pore foams at room temperature exhibiting cubic austenite structure.

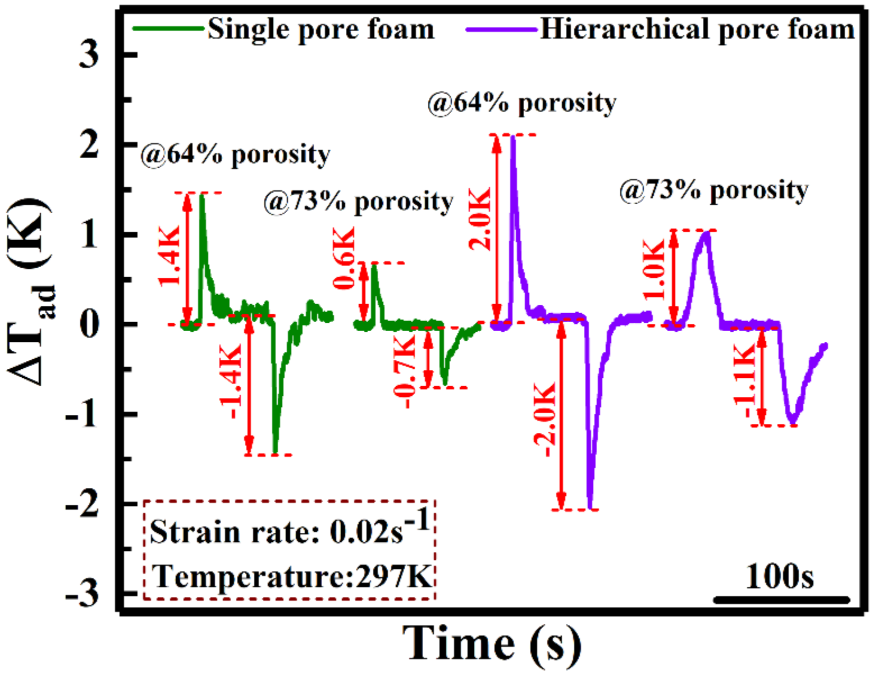

Figure S5. Time-dependence ΔT_{ad} in single-/hierarchical pore architectures Ni-Fe-Ga foams with high porosities 64% and 73% at room temperature.

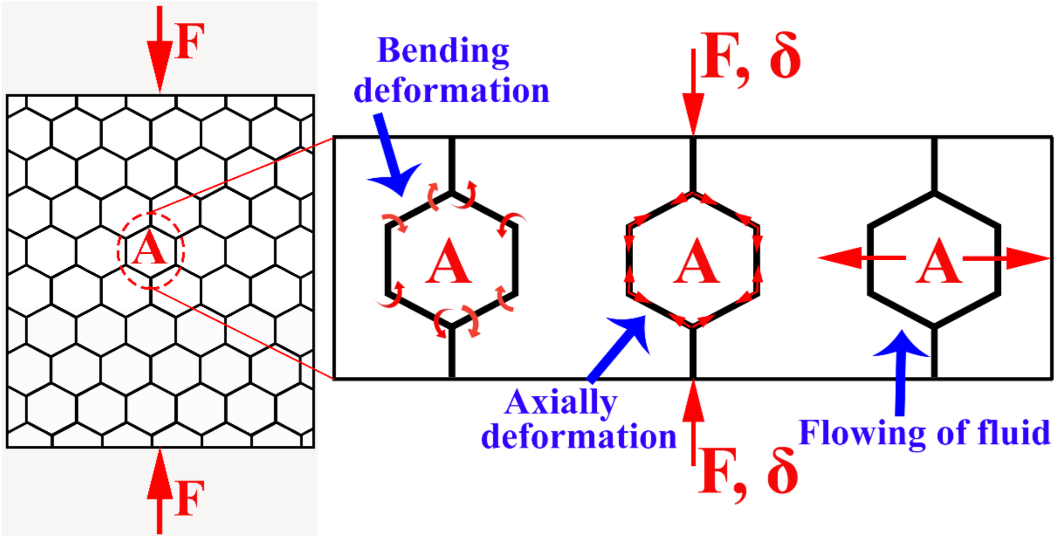

Figure S6. A honeycomb with hexagonal cells represents the open pore foam architecture, which showing the deformation mechanism in simple geometry of foam.

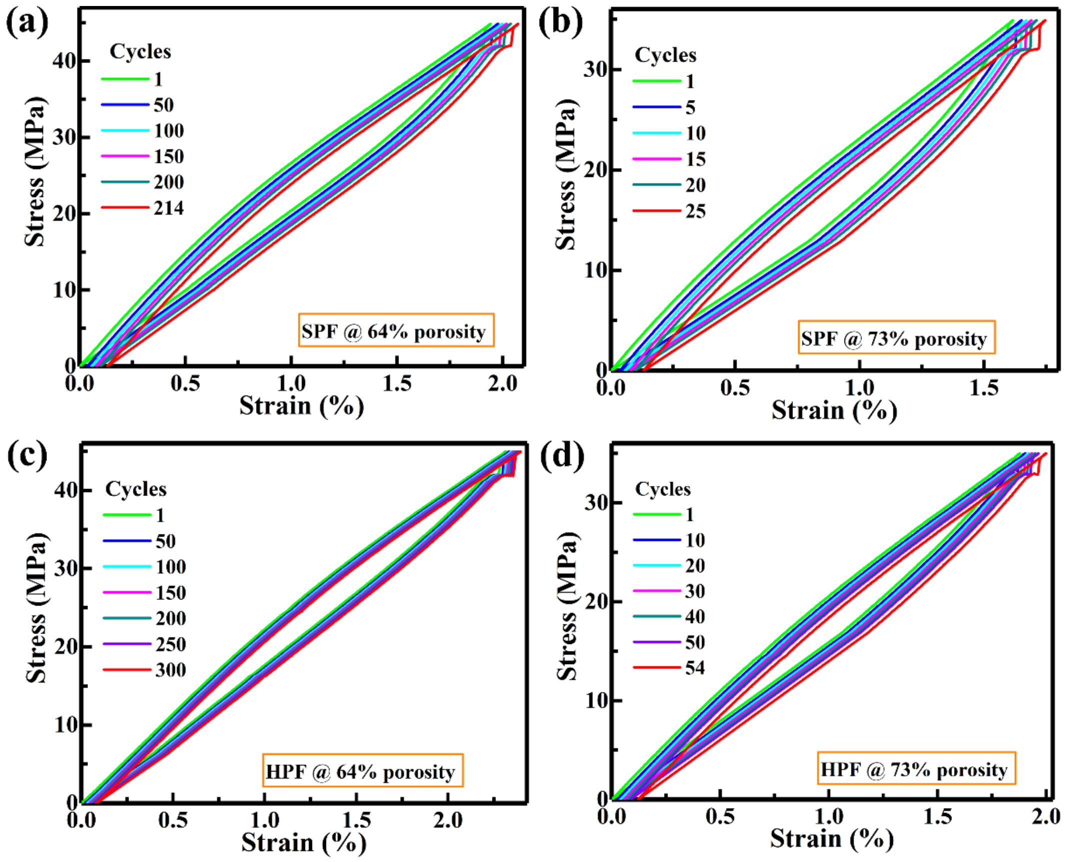

Figure S7. Cyclic stress-strain profiles in single-/hierarchical pore architectures Ni-Fe-Ga foams with high porosities 64% and 73% at room temperature.

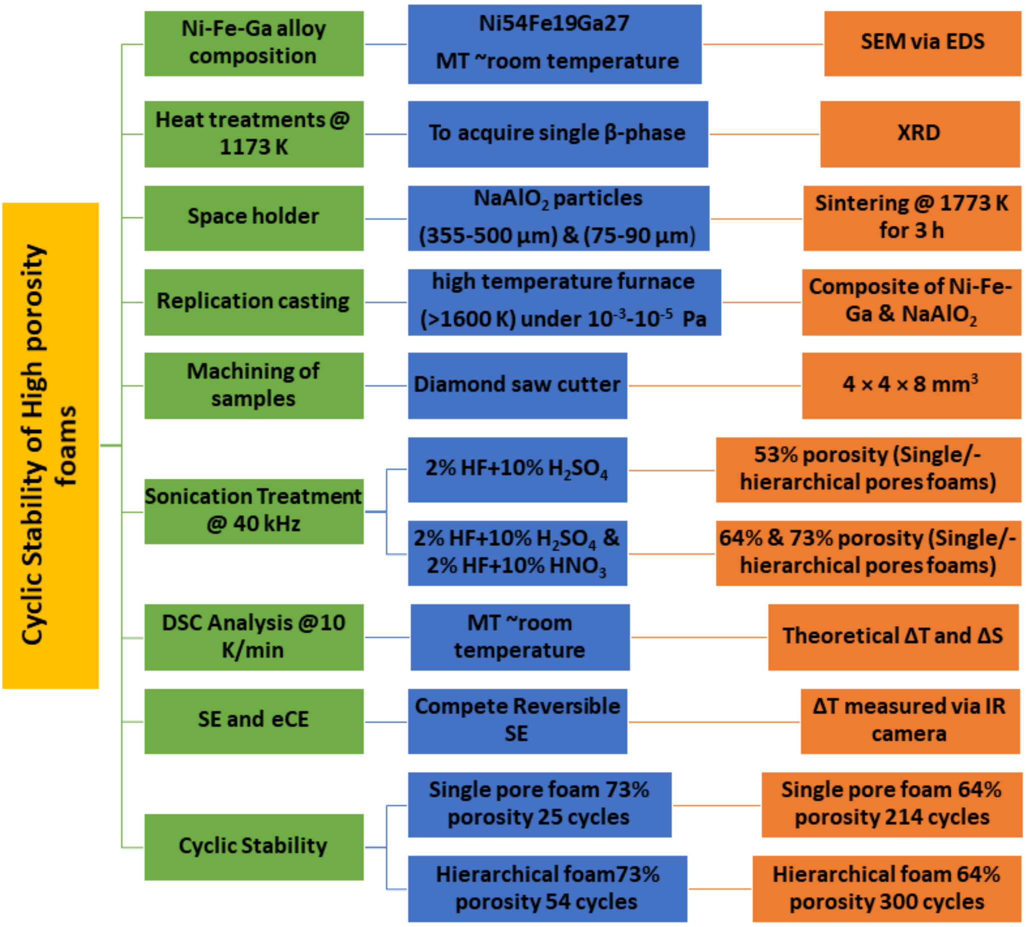

Figure S8. A complete schematic diagram about the large cyclic stability of high porosity Ni-Fe-Ga foams.
